# Oxytocin and social pretreatment have similar effects on processing of negative emotional faces in healthy adult males

**DOI:** 10.3389/fpsyg.2013.00532

**Published:** 2013-08-14

**Authors:** Anna Kis, Kinga Kemerle, Anna Hernádi, József Topál

**Affiliations:** ^1^Psychobiology Research Group, Research Centre for Natural Sciences, Institute of Cognitive Neuroscience and Psychology, Hungarian Academy of SciencesBudapest, Hungary; ^2^Department of Ethology, Eötvös UniversityBudapest, Hungary; ^3^Department of Ecology, Szent István UniversityBudapest, Hungary

**Keywords:** oxytocin, social stimuli, face processing, negative emotion, memory

## Abstract

Oxytocin has been shown to affect several aspects of human social cognition, including facial emotion processing. There is also evidence that social stimuli (such as eye-contact) can effectively modulate endogenous oxytocin levels. In the present study we directly tested whether intranasal oxytocin administration and pre-treatment with social stimuli had similar effects on face processing at the behavioral level. Subjects (*N* = 52 healthy adult males) were presented with a set of faces with expressions of different valence (negative, neutral, positive) following different types of pretreatment (oxytocin—OT or placebo—PL and social interaction—Soc or no social interaction—NSoc, *N* = 13 in each) and were asked to rate all faces for perceived emotion and trustworthiness. On the next day subjects' recognition memory was tested on a set of neutral faces and additionally they had to again rate each face for trustworthiness and emotion. Subjects in both the OT and the Soc pretreatment group (as compared to the PL and to the NSoc groups) gave higher emotion and trustworthiness scores for faces with negative emotional expression. Moreover, 24 h later, subjects in the OT and Soc groups (unlike in control groups) gave lower trustworthiness scores for previously negative faces, than for faces previously seen as emotionally neutral or positive. In sum these results provide the first direct evidence of the similar effects of intranasal oxytocin administration and social stimulation on the perception of negative facial emotions as well as on the delayed recall of negative emotional information.

## Introduction

Despite its complexity (Richerson and Boyd, 1998), recent studies have provided substantial insights into the neurohormonal mechanisms underlying human sociality (Skuse and Gallagher, 2009). Oxytocin, which is—in evolutionary terms—a remarkably conservative non-apeptide, plays a particularly prominent role in the modulation of social life across mammalian taxa (Yamasue et al., 2012). This neurohormone for example has been shown to regulate social contact (Bales and Carter, 2003), pair bonding (Insel and Shapiro, 1992), maintenance of monogamous relationships (Scheele et al., 2012) and parental care (Olazábal and Young, 2006). More importantly, increasing body of evidence supports the notion that oxytocin is specifically involved in the regulation of human social cognition (Lee et al., 2009). It has been shown to reduce fear responses to social stimuli (Kirsch et al., 2005) through the attenuation of amygdala activation (Domes et al., 2007a), that encourages social approach, affiliation and complex social phenomena, such as trust (Kosfeld et al., 2005; Baumgartner et al., 2008) or generosity (Zak et al., 2007; Barraza et al., 2011).

One of the striking features of human sociality is that face perception plays a critical role in modulating social interactions. Faces are highly important stimuli for humans, as they convey vital information about the interactants' identity as well as their mental and emotional states (gender, age, familiarity, intention etc.). Although face perception is not a completely automatic process but also requires attentional resources (Jung et al., 2012), many argue that face recognition is mediated by a specialized system in the human brain (Haxby et al., 2002). This specialization is also supported by the selective impairment of face recognition observed in patients suffering from prosopagnosia (Farah, 1996). Recent evolutionary accounts of the mechanisms behind human face recognition suggest that face perception is essential for distinguishing defectors from co-operators as well as for regulating our social relations (e.g., Kovács-Bálint et al., in press). Facial characteristics thus convey extremely important information for humans in everyday life, people can form an opinion of the trustworthiness and cooperativeness of others purely on the basis of facial photos (Verosky and Todorov, 2010).

Not surprisingly, face perception (or at least some aspects of it) is also mediated by the neurohormone oxytocin that for example increases gaze to the eye region of human faces (Guastella et al., 2008a; Andari et al., 2010) and perceived facial trustworthiness and attractiveness (Theodoridou et al., 2009). Oxytocin has also been shown to enhance facial perception and recognition in humans (Savaskan et al., 2008; Rimmele et al., 2009) as well as to improve emotion recognition from faces (Domes et al., 2007a) especially in subjects suffering from difficulties in understanding and regulating emotions (alexithymia: Steiner, 2008). In line with these results Guastella and his colleagues also reported positive effects of intranasal oxytocin on adult male humans' social memory (Guastella et al., 2008b). In their study human faces showing happy, angry or neutral expressions were presented to participants who received either intranasal oxytocin or placebo. They found that on the following day participants who had been given oxytocin provided more “remember” responses when the faces were previously seen with a happy, rather than an angry or neutral expression.

Another line of research addresses the role which social stimuli may play in the production of oxytocin. Increasing evidence suggest that the release of oxytocin can be effectively induced by short-term sensory interactions including tactile (touch, stroking), visual (eye contact), auditory, and olfactory stimuli (Feldman et al., 2010; Gordon et al., 2010; Kenkel et al., 2012). Thus, it seems that social stimuli and the release of central oxytocin in response to these stimuli constitute a positive feedback loop (Uvnas-Moberg, 1998). Although the role that social stimulus-induced peripheral autonomic changes (e.g., heart and gut reactions) play in oxytocin release is not yet fully understood (Churchland and Winkielman, 2011), there is evidence that positive social interactions can cause changes in behavior through the release of endogenous oxytocin (e.g., in a monetary game, Morhenn et al., 2008). These feed-forward effects might furthermore be mediated by autoreceptors on oxytocin neurons (Freund-Mercier and Stoeckel, 1995).

Importantly, however, it has never been directly tested whether these social stimuli have the same effect as intranasal oxytocin administration at the behavioral level. Therefore, the present study was developed with the aim of combining a behavioral task (face perception and recognition) with four different types of pretreatment; oxytocin/placebo administration and social interaction/no social interaction. We hypothesized that both oxytocin and pretreatment with social interaction would result in more positive emotion and trustworthiness ratings of facial expressions. We further hypothesized that both oxytocin and pretreatment with social interaction would enhance the encoding and/or retrieval of social memory.

## Method

### Subjects

Fifty six healthy, Hungarian, young adults (range: 18–30 years, mean age ± SD: 23.02 ± 3.32) participated in the study on a voluntary basis, that were not selected to be from a specific subrace of Caucasian type. In order to avoid confounding effects of hormone changes across the menstrual cycle (cf. Domes et al., 2007b; Guastella et al., 2008a) all participants were male. Upon arrival participants filled out a brief self-report questionnaire to exclude psychiatric disorders, substance dependence and traumatic brain injury (exclusion criteria were based on Guastella et al., 2008b). Before the experiment participants also filled out the Positive and Negative Affect Scale—PANAS (Watson et al., 1988) to assess general mood (from now on PANAS affect score) and current mood (from now on PANAS state score). In addition to the assessment of subjects' initial mood, participants were asked to fill out the PANAS state questionnaire three more times; after the learning phase as well as both before and after the test phase (see below for more details). Thus, we could track potential changes in participant's current mood throughout the experiment.

We should note that four participants were excluded from the analyses due to their extreme negative self-reported current mood. The criterion for exclusion was based on the assessment of subjects' “initial mood” (measured by PANAS state questionnaire); those who reached higher than 20.2 Negative Affect Score, that is a score greater than the mean + SD of the PANAS state score according to the standards of Watson et al. (1988), were left out from the analysis.

The remaining participants (*N* = 52) were randomly assigned to one of four treatment groups (oxytocin—OT, placebo—PL, social interaction pretreatment—Soc, no social interaction pretreatment—NonSoc, 13 in each) so that these did not differ significantly in age [ANOVA, *F*_(3, 51)_ = 1.220, *p* = 0.313]. No difference was found among the four groups with respect to their PANAS affect scores [ANOVA, *F*_(3, 51)_ = 0.663, *p* = 0.579] either.

All tests were conducted between 2 p.m. and 6 p.m. in order to control for oxytocin level variation due to daily cycle (Forsling et al., 1998). Participants were instructed to abstain from alcohol and caffeine on the day of the procedure and food and drink (except water) 2 h before the test. All subjects provided written informed consent before participation. Ethical approval was obtained from the National Psychological Research Ethics Committee (Ref. No. 2011/13).

### Pretreatment

**Figure 1:**
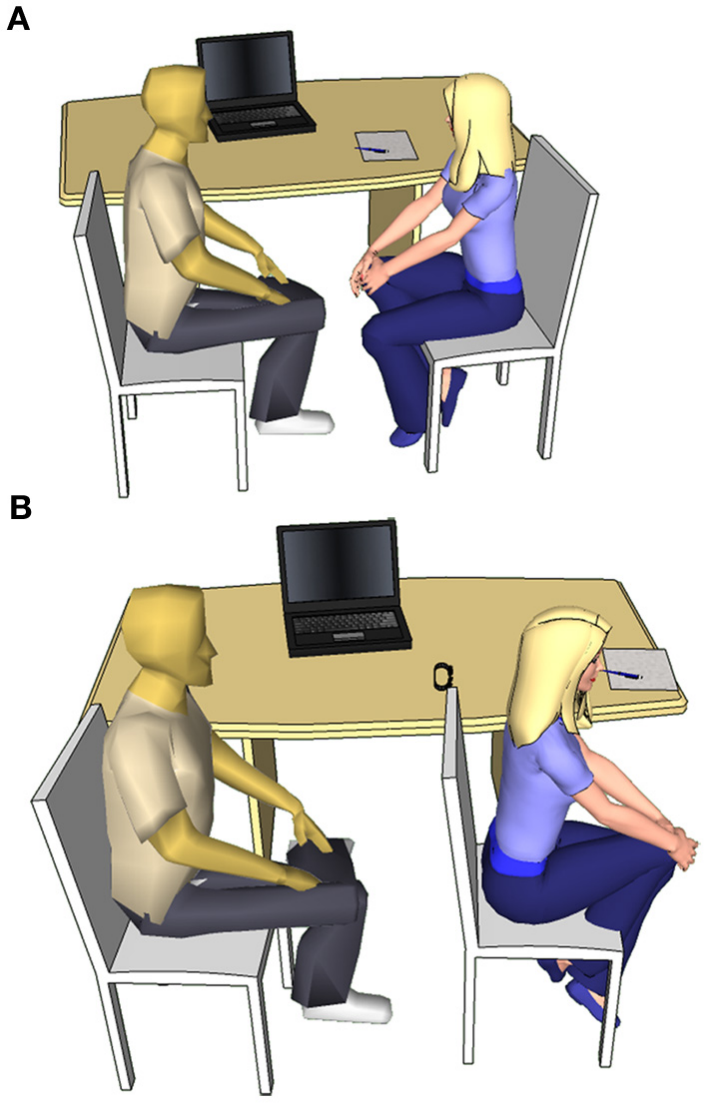
**Social interaction (A) and No social interaction (B) pretreatment setup**.

Half of the participants were told at the beginning of the study that they would receive oxytocin treatment (in order to avoid limitations arising from participant expectations in double-blind placebo-controlled design, cf. Kaptchuk, 2001; Colagiuri, 2010) and were then randomly assigned in a single-blind manner to receive either 24 international units (IU) of oxytocin, commercially available Syntocinon-Spray (OT, *N* = 13) or placebo (PL, *N* = 13), isotonic natriumchlorid 0.9% solution (cf. Savaskan et al., 2008) followed by a 40-min waiting period in a quiet room isolated from social stimuli, that is necessary for the central oxytocin levels to reach a plateau. (There is a tacit assumption in the literature that intranasal administration of oxytocin enables direct access of the peptide to the central nervous system (CNS), thus providing a useful method for studying the specific effects of this neuropeptide on the regulation of behavior. This is based in the work by Born et al. (2002) showing that after intranasal administration of melanocortin, vasopressin and insulin to human subjects the concentration of these peptides was elevated in the CNS., More recently Neumann et al. (2013) provided more direct evidence for an association between intranasal administration of oxytocin and the increased level of this neuropeptide in both the hippocampus and amygdala.) The other half of our subjects were randomly assigned to receive either social interaction (Soc, *N* = 13) or no social interaction (NSoc, *N* = 13) pretreatment (Figure 1). Subjects merely received instructions they had to follow during pretreatment, but no other information. The Social interaction consisted of a 6-min session alternating *phase 1* when the subject was instructed to make eye-contact with a female experimenter for 30 s and *phase 2* when the experimenter took the subject's wrist pulse (tactile stimuli) for 30 s. Every 30 s a helper invisible to the subject indicated the end of the phase by knocking. The NSoc pretreatment consisted of a 6-min session alternating *phase 1* when the subject was instructed to focus on the back of the experimenter for 30 s and *phase 2* when the subject was wearing a pulsometer on the wrist; the end of each phase was indicated by the knocking of the experimenter. In both cases the experimenter noted the pulse of the subjects in order to see if any of them showed a stress response as a result of the pre-treatment compared to the baseline due to the unnaturally prolonged eye-contact and social interaction (c.f. Argyle and Dean, 1965). However, no such response could be observed as subjects' pulse remained stable in the NSoc group [*t*_(12)_ = 0.131, *p* = 0.898] and even decreased in the Soc group [*t*_(12)_ = 3.107, *p* = 0.009] that is consistent with the cardio-vascular effects of oxytocin (Light et al., 2005; Gutkowska and Jankowski, 2008). Soc and NSoc pretreatments were immediately followed by the learning phase without a waiting period.

### Learning phase

Stimuli used in the experiment were selected from the Radboud Faces Database (Langner et al., 2010; www.rafd.nl) that contained colored photos taken against uniform white background of Caucasian (Dutch) adult models wearing black t-shirts, having no facial hair and wearing no glasses, makeup or jewelry. The database exhibited some “natural,” and in the present study uncontrolled, variability in facial morphology and skin tone within the Caucasian ethnic group as our model faces were from both Nordic and Atlanto Mediterranean types. One third of the images were female and two third were male faces. Only face images taken from a 90° camera angle were shown and the models' eyes were always directed straight ahead. Each model was presented with one emotional expression (happy/neutral/angry/fearful) only. Stimuli were presented from a visual angle of 28.07° × 19.85° under a viewing distance of 40 cm.

Following pretreatment all four groups of subjects were presented with 36 images of human faces; 12-12-12 showing positive (happy), neutral or negative (angry/fearful) emotional expression for 2 s each, on a computer screen. The order of presentation was a fixed pseudorandom sequence where the models of different gender as well as the different emotional expressions were intermixed (see Supplementary material). Participants were asked to rate the degree to which each model seemed trustworthy and appeared to show positive facial emotion using a 1–9 Likert scale (1—not at all, 9—definitely); the order of questions was fixed (first: trustworthiness: second: emotion). Although it has been argued (Todorov, 2008) that trustworthiness judgments are an extension of emotional judgments, we decided to include both scales as some emotions have been reported to correlate with trustworthiness while others not (Winston et al., 2002). Furthermore, there is no direct evidence suggesting that oxytocin affects trustworthiness and emotion ratings in the same way. At the end of the learning phase participants filled out the PANAS state questionnaire for a second time in order to track mood changes.

### Test phase

Subjects returned on the following day at around the same time of the day (±60 min). Before the test itself all participants were asked to fill out the PANAS state questionnaire. Participants were then presented with 36 images of human faces with neutral expression. Half of the images were faces that had also been shown on the previous day, and from these now neutral faces 6-6-6 had formerly been positive, neutral and negative respectively. Thus, formerly neutral faces were identical to the ones that had been shown in the learning phase, while on the pictures of formerly positive and negative facial expressions the same persons as in the learning phase were presented, with novel (neutral) facial expression. Participants, again, had to rate all faces on a nine point Likert scale; both the extent to which the given face expressed positive emotion and the trustworthiness of faces (1—not at all, 9—definitely). Furthermore, they were asked if they had seen the same person on the previous day (Yes/No). After the test participants filled in the PANAS state questionnaire once more. Afterwards participants in the OT/PL group were given the information that the study was placebo controlled and were asked to make a guess about treatment received. No difference could be observed between groups in their guesses [χ^2^-test, χ^2^_(1)_ = 0.024, *p* = 0.875]. At the end of the session the experimenter asked the participants in the OT/PL group about any side effects they may have noticed; but no side effects were reported in any of the two groups.

### Data analysis

In order to compare the PANAS affect scores between the four pretreatment groups we used an analysis of variance (ANOVA). PANAS state scores were analyzed using generalized estimating equations (GEE) method to assess the effects of different factors: pretreatment (OT, PL, Soc, NSoc), phase (before or after image presentation) and test occasion (1st or 2nd day). Pearson correlations were used to check the relatedness of the Emotion and Trustworthiness rating scales both at the group and at the individual level. Individual correlation coefficients were compared to chance level (one-sample *t*-test) and across pretreatment groups (ANOVA). We also used GEE models to identify potential factors (pretreatment, test occasion and type of facial expression), with significant effects on the Trustworthiness and Emotion ratings. Follow up tests were run to directly compare Trustworthiness and Emotion ratings between OT vs. PL and Soc vs. NSoc groups (independent *t*-tests) for the three stimuli types separately. Additionally and item analysis was carried out comparing OT vs. PL and Soc vs. NSoc groups by averaging subjects' answers for each image separately (paired samples *t*-tests). Recognition memory (the percentage of correct judgments of whether a face had been seen or not on the previous day) was compared between OT vs. PL and Soc vs. NSoc groups (independent *t*-tests) both on the whole dataset and for different stimuli types separately. In the test phase the four pretreatment groups were also compared with respect to Trustworthiness and Emotion ratings of formerly negative, neutral and positive stimuli (independent *t*-tests). A within-subject analysis comparing Trustworthiness and Emotion ratings of novel faces and faces previously shown with negative, neutral and positive emotions was also run (ANOVA) for the four pretreatment groups separately.

## Results

### Evaluation of positive and negative affect

PANAS state scores were not affected by pretreatment, and the scores obtained before and after the learning/test phases did not differ significantly (GEE, all *p* > 0.1). However, subjects on the second day reported a more negative mood [χ^2^_(1)_ = 5.963, *p* = 0.015]. No interaction was found among the factors (all *p* > 0.1).

### Overall analysis of trustworthiness and emotion ratings given by participants

**Figure 2:**
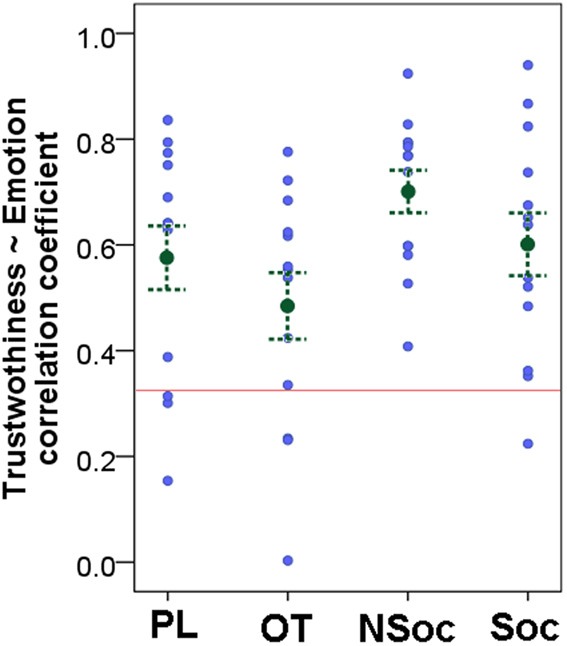
**Correlatedness of Trustworthiness and Emotion ratings in the four groups.** Mean ± SE; gray dots show individual data; red line indicates significance level.

A Pearson correlation (taking the average scores from all participants of the respective rating for each image) showed that Trustworthiness and Emotion ratings were highly correlated in all four groups (OT: *r* = 0.693, *p* < 0.001; PL: *r* = 0.840, *p* < 0.001; Soc: *r* = 0.933, *p* < 0.001; NSoc: *r* = 0.898, *p* < 0.001). This high correlatedness held for the happy (*r* = 0.646, *p* = 0.023), neutral (*r* = 0.912, *p* < 0.001) and angry (*r* = 0.922, *p* = 0.009), but not for fearful faces (*r* = −0.526, *p* = 0.284). However, individual correlation coefficients (based on Trustworthiness and Emotion ratings of all images) varied greatly, and were not significant for all subjects (Figure 2) suggesting that subjects' strategies might differ in assessing these two ratings. At the group level the correlation coefficients were higher than zero [*t*_(51)_ = 20.086, *p* < 0.001] and the four groups did not differ with regard to the correlatedness of the Trustworthiness and Emotion ratings, although there was a trend for lower correlatedness in the OT and Soc groups as compared to the PL and NSoc groups respectively [*F*_(3, 48)_ = 2.489, *p* = 0.072].

A GEE Model revealed that trustworthiness ratings were influenced by stimulus type [negative/neutral/positive; χ^2^_(2)_ = 103.773, *p* < 0.001], but the main effects of pretreatment (OT/PL/Soc/NSoc) and test occasion (learning,) were statistically not significant (all *p* > 0.1).

**Figure 3:**
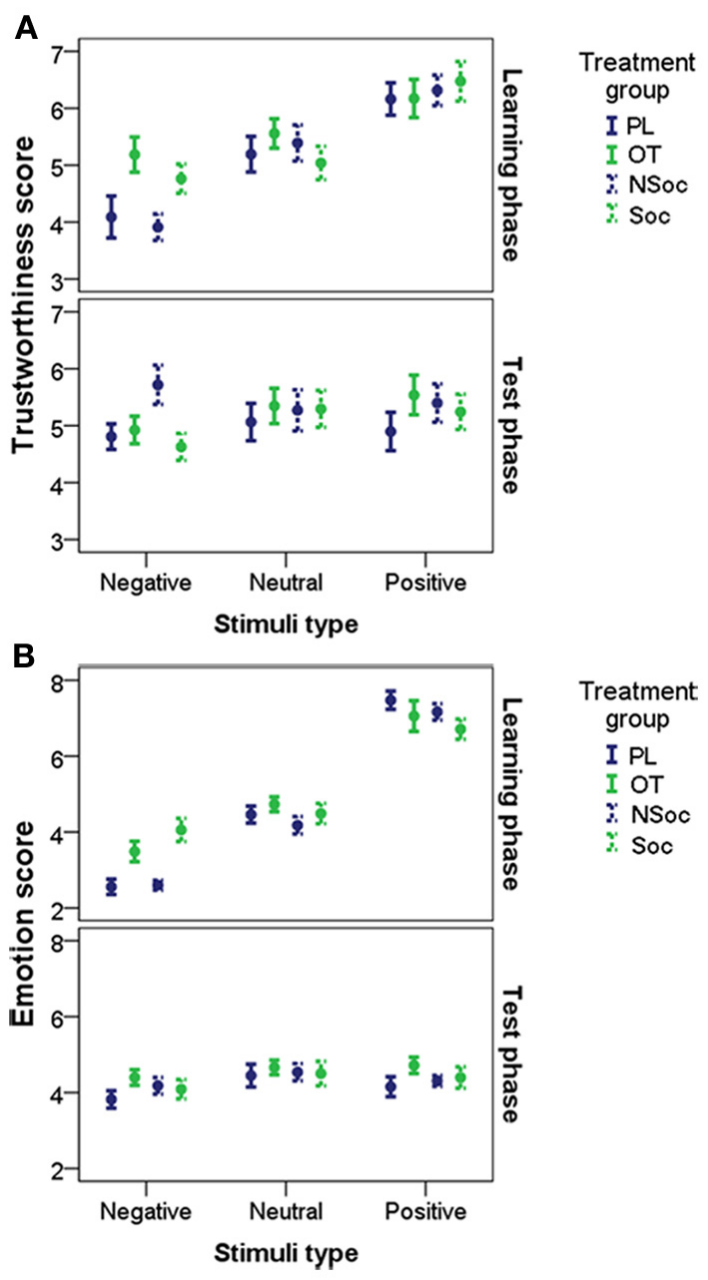
**Trustworthiness (A) and Emotion (B) scores (mean ± SE) in the OT/PL and Soc/NSoc groups for negative, neutral and positive faces in the learning and the test phases**.

A significant interaction was found between stimulus type × test occasion [χ^2^_(2)_ = 101.528, *p* < 0.001]. The interaction between stimulus type, × pretreatment × test occasion was also significant [χ^2^_(6)_ = 54.864, *p* < 0.001]. This suggests that faces with negative emotional expression (but not the others) were rated more positively by subjects in the OT and Soc pretreatment groups than in the PL and NSoc groups in the learning phase, but not in the test phase (Figure 3A).

A GEE Model revealed that emotion ratings were influenced by stimulus type [negative/neutral/positive; χ^2^_(2)_ = 289.971, *p* < 0.001] and test occasion [learning/test phases; χ^2^_(1)_ = 31.254, *p* < 0.001] but the main effect of pretreatment [OT/PL/Soc/NSoc; χ^2^_(3)_ = 5.464, *p* = 0.141] was not significant.

However, the interaction between stimulus type and pretreatment was significant [χ^2^_(6)_ = 15.118, *p* = 0.019). In addition, there was a significant stimulus type × test occasion interaction [χ^2^_(2)_ = 403.546, *p* < 0.001]. More importantly, the interaction between stimulus type × pretreatment × test occasion was also significant [χ^2^_(6)_ = 26.613, *p* < 0.001]. Thus, it seems that faces with negative emotional expression (but not the others) were rated more positively by subjects in the OT and Soc groups than in the PL and NSoc groups in the learning phase, but not in the test phase (Figure 3B).

### Differences in trustworthiness and emotion ratings between OT vs. PL and soc vs. nsoc groups

#### Learning phase

Subjects in the OT group rated negative emotional faces more positively than subjects in the PL group both with respect to trustworthiness [two-sample *t*-test, *t*_(24)_ = 2.279, *p* = 0.032] and perceived emotion [*t*_(24)_ = 2.768, *p* = 0.011]. However, no such differences were found in case of faces with neutral and positive emotional expression (*p* > 0.1 in all cases, for both trustworthiness and emotion). An item analysis yielded similar results: Trustworthiness: OT > PL [*t*_(35)_ = 4.362, *p* < 0.001]; Emotion: OT > PL [*t*_(35)_ = 2.246, *p* = 0.031].

Subjects in the Soc group also rated negative emotional faces more positively than subjects in the NSoc group both with respect to trustworthiness [*t*_(24)_ = 2.474, *p* = 0.021] and perceived emotion [*t*_(24)_ = 4.390, *p* < 0.001]. No such difference was found in case of faces with neutral and positive emotional expression (*p* > 0.1 in all cases, for both trustworthiness and emotion). An item analysis yielded similar results: Trustworthiness: Soc ≥ NSoc [*t*_(35)_ = 1.886, *p* = 0.068]; Emotion: Soc > NSoc [*t*_(35)_ = 2.953, *p* = 0.006].

#### Test phase

No differences were found in the percent of correct judgments about whether a face had been seen or not on the previous day neither between OT and PL nor between Soc and NSoc (two-sample *t*-test, *p* > 0.1 in all cases). This is also true for the analysis of faces previously seen with negative, neutral and positive facial expressions separately (*p* > 0.1 in all cases). Moreover, treatment groups (OT vs. PL and Soc vs. NSoc) did not differ in their trustworthiness and emotion ratings either (*p* > 0.1 in all cases). (Note that each face in the test phase displayed a neutral expression.)

**Figure 4:**
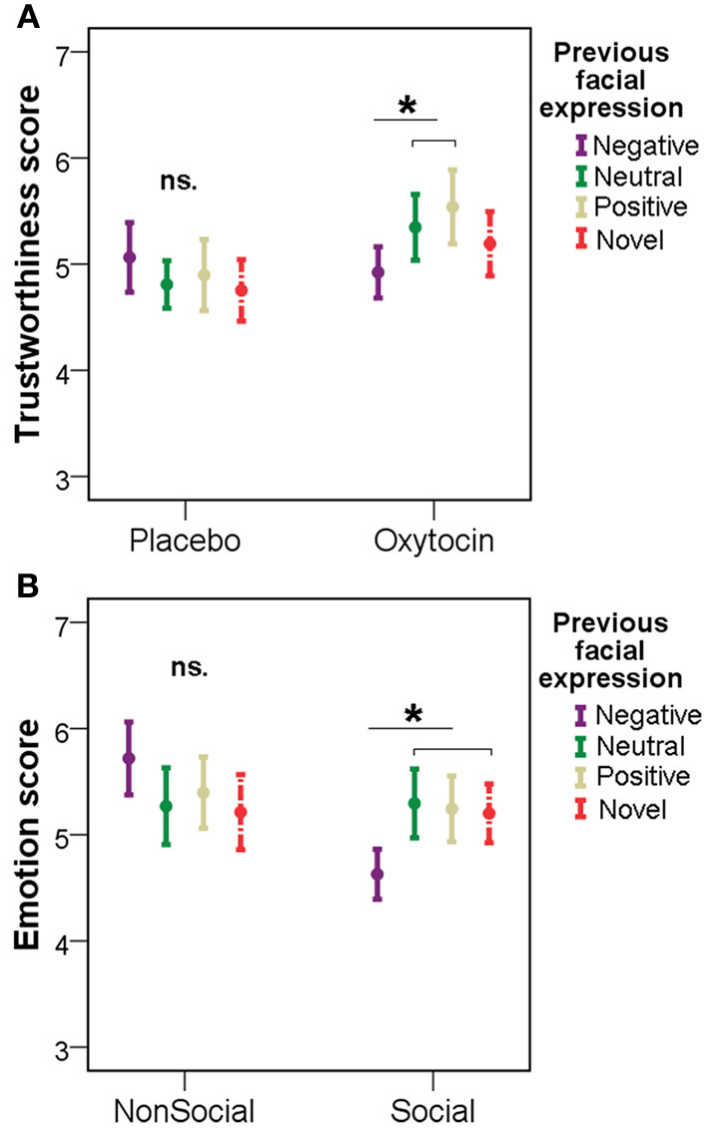
Trustworthiness scores (mean ± SE) for faces that had previously been shown with positive/negative/neutral emotional expressions in the PL/OT (A) and NSoc/Soc (B) groups. ^*^p < 0.05.

Interestingly, however, when studying how the participants in the different treatment groups rated novel faces and those that had previously been shown with positive/negative/neutral emotional expressions, we found differential effects of pretreatments. Namely, subjects in the OT group gave lower trustworthiness scores for neutral faces previously seen with negative expressions, than for previously neutral and positive faces [repeated measures ANOVA, *F*_(3, 36)_ = 5.025, *p* = 0.005; LSD *post-hoc* tests: negative vs. neutral: *p* = 0.031, negative vs. positive: *p* = 0.014, negative vs. novel: *p* = 0.197, neutral vs. positive: *p* = 0.187, neutral vs. novel: *p* = 0.183, positive vs. novel: *p* = 0.160]. No such difference could be observed in the PL group between faces previously seen with negative/neutral/positive facial expression and novel faces [*F*_(3, 36)_ = 1.120, *p* = 0.354] (Figure 4A).

Similarly, participants in the Soc group gave lower trustworthiness scores for previously negative faces, than for previously neutral/positive and novel faces [repeated measures ANOVA, *F*_(3, 36)_ = 5.036, *p* = 0.013; LSD *post-hoc* tests: negative vs. neutral: *p* = 0.017, negative vs. positive: *p* = 0.023, negative vs. novel: *p* = 0.019, neutral vs. positive: *p* = 0.796, neutral vs. novel: *p* = 0.588, positive vs. novel: *p* = 622]. In the NSoc group, however, participants' trustworthiness ratings were not affected by the previously seen emotions [*F*_(3, 36)_ = 2.843, *p* = 0.100] (Figure 4B).

Subjects' emotion ratings, unlike trustworthiness ratings, were not affected by previous facial expressions in the OT, Soc and NSoc groups (*p* > 0.05 in all cases). Participants in the PL group, however, rated previously negative emotional faces more negatively than previously neutral and novel faces [*F*_(3, 36)_ = 5.596, *p* = 0.003; LSD *post-hoc* tests: negative vs. neutral: *p* = 0.004, negative vs. positive: *p* = 0.113, negative vs. novel: *p* = 0.010, neutral vs. positive: 0.066, neutral vs. novel: *p* = 0.231, positive vs. novel: 0.416].

## Discussion

Our study provides the first experimental evidence that exogenously administered oxytocin and preexposure to social stimuli have similar effects on the perception of negative but not positive and neutral facial emotions in adult human male subjects. That is, after having received intranasal administration of oxytocin or social stimulation (via eye contact and touching the skin) participants rated negative emotional faces more positively than subjects in the control groups. These results provide empirical evidence to support the widely held but unjustified notion that pretreatment with oxytocin and social interaction have similar behavioral effects on adult male humans. We should note, however, that although this finding raises the possibility that the effects of both types of pretreatment may be mediated by increases in central oxytocin, we have no evidence to suggest the very same underlying neuro-physiological mechanism.

Although in the current study we used an all-male sample with a fixed-order stimuli presentation (making it possible that the results are subject to order effects), our findings seem to confirm previous reports suggesting that intranasal administration of oxytocin may have a modulating effect on face perception in humans (e.g., Guastella et al., 2009); furthermore as all groups viewed the same set of stimuli, even if order had an effect of subjects' ratings, it should have affected all groups in the same way, thus the differences we found can only be attributed to the pretreatments used. A further limitation of our study is that we used an isotonic natriumchlorid 0.9% solution as placebo, but as it has been used previously in similar studies (cf. Savaskan et al., 2008) and participants were unable to find out whether they received oxytocin or placebo, we do not think that this could have biased our results. Moreover, assuming that human social interactions have the potential to facilitate the release of oxytocin in the brain (Gordon et al., 2010) through a positive feedback loop (Zak et al., 2005), our results are in line with previous findings that have demonstrated correlations between social stimulation and those behavior changes [generosity (Morhenn et al., 2008) or monetary sacrifice (Morhenn et al., 2008) in the trust game] that are supposedly associated with an increase in endogenous oxytocin levels.

In line with previous arguments (e.g., Todorov, 2008) we found that subjects' rating on the Trustworthiness and Emotions scales were highly correlated, and although high inter-individual variation could be observed OT or Soc pretreatment had no effect on this relatedness. One factor that might have contributed to this relatedness is that subjects had to provide the two rating at the same time, thus their judgments were most probably not independent from each other.

Importantly, our results show a selective effect of both OT and Soc pretreatment on the perception of different facial emotions (during the learning phase). This is consistent with the notion that oxytocin modulates neural circuitry for social cognition through decreasing the activation of the amygdala (Domes et al., 2007a). This brain region, responsible for the regulation of negative emotions and fear responses (Phan et al., 2002; Gläscher et al., 2004), is rich in oxytocin receptors (Huber et al., 2005), and this finding likely explains why oxytocin and social pre-treatments only had an effect on the perception of negative emotional faces. Further studies should reveal how the oxytocin system interacts with other factors (e.g., facial morphology—Oosterhof and Todorov, 2008; other race effect—O'Toole et al., 1994; Sheng et al., 2012) that influence subjects' judgments about faces.

In addition to short-term effects within the time window of the presumed uptake of exogenous oxytocin (face perception), long-term effects (face recognition memory) after elimination of intranasal oxytocin from CNS have also been reported in the literature. However, in contrast to previous results showing that oxytocin enhances the recognition of faces (Guastella et al., 2008b; Savaskan et al., 2008; Rimmele et al., 2009), in our study we could not find any differences between OT and PL as well as between Soc and NSoc groups in terms of face-recognition. This might be attributed to methodological differences compared to previous experiments. Namely, in the (Savaskan et al., 2008) study subjects received oxytocin administration *after* (and not before) the “acquisition” phase; (Guastella et al., 2008b; Rimmele et al., 2009) used a remember/know paradigm (while our subjects only gave yes/no answers). Furthermore, Rimmerle used a surprise memory test (as opposed to our setup where subjects were told after the learning phase that they would participate in a recognition task on the next day).

Another possible explanation is that in the present study the recognition-improving effect of oxytocin was masked by an internal context change (difference in the level of oxytocin during the learning and the recalling phase) that has been shown to decrease memory performance (Smith and Glenberg, 1978). In experiments testing memory performance internal context change is usually defined as a mood change (Davies, 1986). In our experiment oxytocin administration and social stimulation did not affect subjects' mood (as measured by the Positive Negative Affectivity Scale before and after treatments), however, subjects on the second day reported a more negative mood compared to the first day, suggesting changes in internal context. This was probably so because of “test anxiety” (Spielberger, 1972): in the test phase participants might have recognized the recall task as a cognitively demanding situation which could lead to increased anticipatory and/or situational anxiety and thus might increase participants' negative moods. Internal context changes have been found to influence recall tests but had little or no impact on recognition tests (Davies, 1986), the task that was used in the present study.

Although participants receiving OT vs. PL or Soc vs. NSoc pretreatments did not show different face recognition performance in the test phase (24 h after treatment), we could find some evidence for the long-term effects of both exogenous oxytocin administration and social stimulation on subjects' social memory. Subjects in the OT and Soc groups (unlike in control groups) gave lower trustworthiness scores for those neutral faces that had previously been seen with negative facial expressions, than for faces previously seen as emotionally neutral or positive. These results clearly show a somewhat controversial (note that subjects in OT and Soc group had rated negative emotional faces more positively during the learning phase), yet enduring effect of intranasal oxytocin on social memory (see also Guastella et al., 2008b) and a similar memory-improving effect of social stimulation. Similar results were obtained by Striepens et al. (2012) who found a biased memory toward negative rather than neutral items (from the International Affective Picture System) after oxytocin administration. The authors explain these findings by the fact that according to their fMRI data besides the inhibition of the amygdala, the facilitation of the left insula and an increased functional coupling between the left amygdala, left anterior insula, and left inferior frontal gyrus also occurred. These and our results are also in line with other recent research suggesting that oxytocin is not a magical “trust elixir” (Mikolajczak et al., 2010), and that despite increasing prosocial behaviors, it does not make people blind to negative social stimuli, but on the contrary in some cases it even increases the salience of negative social stimuli (Theodoridou et al., 2013).

Several human psychiatric disorders are characterized by social cognition deficits, including face perception problems, such as autism (Baron-Cohen et al., 2001), schizophrenia (Loughland et al., 2002) or fragile X syndrome (Garrett et al., 2004). Oxytocin has been suggested to be an effective treatment for such social disorders (Heinrichs and Gaab, 2007; Hollander et al., 2007); but see Bartz et al. (2011) for contradictory findings. Our results demonstrating a similar effect of exogeneous oxytocin and social stimulation widen the perspectives of these interventions suggesting that behavioral therapy involving positive social stimuli might be a comparably successful methodological approach. Needless to say, much more research is necessary to determine the extent to which the effect of social stimulation is equivalent to that of oxytocin administration.

In summary, our findings provide new and important insights into how an evolutionarily conservative nanopeptide, the oxytocin, and certain social interactions can affect highly adaptive human behaviors such as face perception and social memory formation.

## Conflict of interest statement

The authors declare that the research was conducted in the absence of any commercial or financial relationships that could be construed as a potential conflict of interest.
